# Carbon Nanotube Reinforced Natural Rubber Nanocomposite for Anthropomorphic Prosthetic Foot Purpose

**DOI:** 10.1038/s41598-019-56778-0

**Published:** 2019-12-27

**Authors:** Rasaq Olawale Medupin, Oladiran Kamardeen Abubakre, Ambali Saka Abdulkareem, Rasheed Aremu Muriana, Asipita Salawu Abdulrahman

**Affiliations:** 1 The Federal Polytechnic, P. M. B. 55, Department of Mechanical Engineering, Bida, Nigeria; 2 Federal University of Technology, P. M. B. 65, Department of Materials & Metallurgical Engineering, Minna, Nigeria; 3 Federal University of Technology, P. M. B. 65, Department of Chemical Engineering, Minna, Nigeria; 4 Federal University of Technology P. M. B. 65, Nanotechnology Research Group, Centre for Genetic Engineering and Biotechnology, Minna, Nigeria

**Keywords:** Mechanical engineering, Carbon nanotubes and fullerenes

## Abstract

This research is motivated by the desire to restore the quality of life to amputees. The study uses multi-walled carbon nanotube (WMCNT) reinforced natural rubber (NR) polymer nanocomposite (PNC) for prosthetic foot application. The compound formulation was carried out in accordance to a modified procedure described by Hemkaew *et al*. Mixing of the ingredients during vulcanisation was performed according to ASTM D-3182 standard on an open two-roll mill. The various compositions of the nanocomposites (NCs) were cured at a temperature of 150 ± 2 °C and a pressure of 0.2 MPa for 10 minutes in an electrically heated hydraulic press. Mechanical investigation revealed that NR/MWCNT-3 exhibited the highest capacity to withstand tensile and dynamic loading (449.79 MPa). It also showed superior filler distribution and hence improved crystallinity and cross-link. Water absorption test indicated that NR/MWCNT-3 offers optimum dimensional stability at ambient conditions. Moreover, thermogravimetric analysis/differential thermogravimetry (TGA/DTG) showed degradation peaks at 305 °C and 290 °C respectively with temperature range within which the NCs degraded lying between 250 °C and 600 °C. Dynamic mechanical analysis (DMA) revealed that filler incorporation results in higher storage and loss moduli (2000–7500 MPa and 500–1413 MPa respectively). Tan δ curves proved that NR/MWCNT-3 has the highest capacity to dissipate energy through segmental motion. Furthermore, microstructure examination confirmed good filler/matrix adhesion as NR/MWCNT-3 indicated improved interaction; hence higher strength (6.02 MPa) of the NC. Better wear resistance ability can also be reported of the newly developed than existing prosthetic material. It can be deduced that the formulated nanocomposite from MWCNTs for reinforced natural rubber is suitable for the development of the anthropomorphic prosthetic foot.

## Introduction

The last two decades have witnessed intense interests of researchers on the development of polymeric nanocomposites in which one of the dimensions of the filler is nano-sized^1,2^. Researchers from across the world have indicated that the addition of low weight percent carbon nanotubes (CNTs) can result in significant improvement on the mechanical properties of biodegradable polymer composite for biomedical engineering application^2,3^. It has been reported that CNT reinforced rubber system exhibits extremely high level of filler interlocking and trapped rubber even at low loading, 5 phr. The weight ratio of carbon black/carbon nanotubes (CB/CNTs) was reported as 20:5 phr^4^. One of such reports by Oboh *et al*.^5^ exposed some of the challenges associated with CB reinforced rubber system. CB is less desirable for reinforcing materials that are expected to undergo cyclic strain because of its particulate nature.

A recent investigation on diabetes mellitus foot gangrene in western Nigeria revealed an alarming 52.2% rate of amputation and 14.3% mortality rate in a group of diabetic patients with foot ulcers^6^. Centres for Disease Control and Prevention (2015), and Ziegler-Graham *et al*.^7^ reported approximately 1.9 million amputees across the US and roughly 185,000 amputation surgeries annually. About 82% of these amputation surgeries are as a result of peripheral vascular disease and diabetes. An estimated 8,900 children are reportedly amputated annually due to domestic mishap and 6,000 of the reported cases are transfemoral amputation. Walter Reed Army Medical Center is reported to be offering treatment to about 1,000 military amputees, while the Veterans Administration (VA) health care system is home to 40,000 amputees. Western developed countries recorded an alarming rate of lower limbs amputations of about 17.1 amputations per 100,000 populace. And in Spain alone 5,000 such amputation surgeries are carried out annually.

These disturbing statistics emphasize the need for urgent and proactive measures to restore meaningful life to the vast number of people who need artificial limbs. However, Li *et al*.^8^ pinpointed cellular toxicity, carcinogenicity, and hypersensitivity as likely dangers posed by metals such as titanium, vanadium, molybdenum, chromium, cobalt, nickel and aluminum when in prolonged contact with human blood. This viewpoint was corroborated by other researchers who established incidences of untimely change of the devices because of the adverse reactions caused by metal implants^9^. The percentage of subjects who present negative sensitivity to metals is appreciably higher in those with metal-on-metal prostheses than those with metal-on-ultra high molecular weight polyethylene (UHMWPE) according to Brown *et al*.^10^. This is reportedly higher in those with unsuccessful metal-on-metal; hence the need to develop a new material that poses no danger to human health.

The use of CNTs as reinforcing filler in polymer matrix has gained the attention of researchers since the early 1990s when single walled and multi-walled carbon nanotubes (SWCNT and MWCNT) were discovered^11^. The elastic modulus of MWCNT is proximately 1 TPa and its tensile strength is estimated to be 100 GPa. This is more than ten times higher than any industrial fibres. However these theorised mechanical potentials of CNTs are yet to be fully realised^12^. The excellent abrasion and tear resistance properties, coupled with good tensile strength make natural rubber (NR) a good candidate for CNTs host.

Obviously, the ultimate mechanical properties and other functional properties of CNT/polymer nanocomposites are determined through the inherent properties of CNTs. Therefore adequate attention needs to be paid to the problems associated with all the activities of CNTs growth if CNT/polymer composites of high standard will be developed. One major challenge is that of ensuring uniform dispersion of CNTs in the host polymers. This study uses defect-free CNTs as reinforcement in NR matrix to develop a low specific weight, high strength prosthetic material that will help to address the question of restoration of quality of life to amputees in low income sub-Saharan African countries like Nigeria.

## Results and Discussion

### Characterisation of the composites

**Figure 1:**
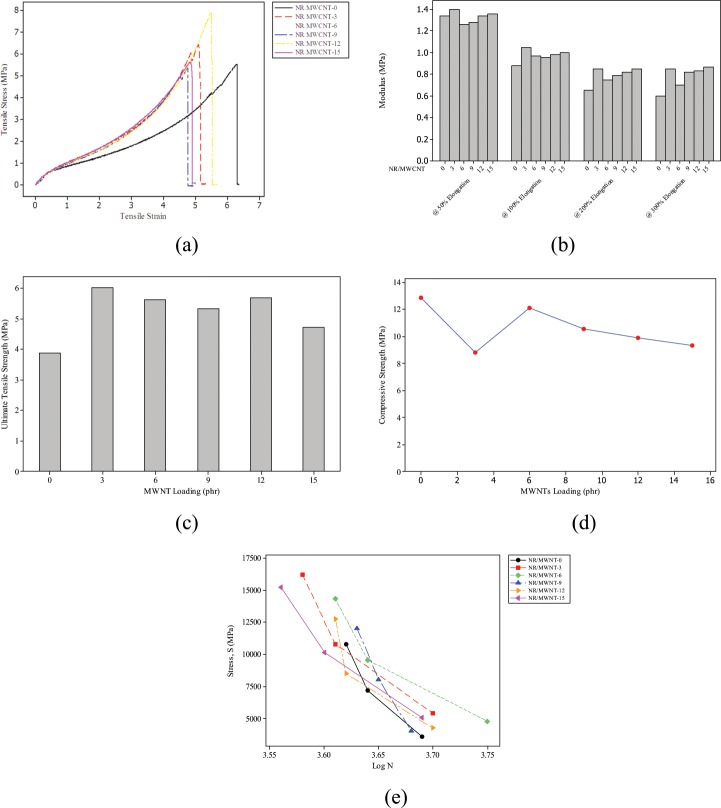
Mechanical Characterisation (**a**) Tress-strain curves (**b**) Tensile dodulus (**c**) Ultimate tensile strength (**d**) Compressive strength (**e**) S-N curves of fatigue test.

The developed composites were characterised to determine their suitability for prosthetic application and the results obtained are hereby presented. The effect of MWCNTs loading on the tensile modulus of the nanocomposites are presented in Fig. 1(a). Results indicated that the tensile modulus of the nanocomposite increases with increase in filler content of the composite. This tends to stiffen and harden the NR vulcanisate thereby reducing its resilience and toughness; impacting negatively on elongation at break^13^. This trend does not hold indefinitely, however, as a point was reached where further increase in filler content no longer have any significant effect on tensile modulus^14^. Resistance to crack propagation is also hampered by unrestricted incorporation of fillers into the matrix which could lead to catastrophic cracks in the long run according to Mahir *et al*.^13^. It is interesting, however, to observe (from Fig. 1(a)) that saturation point was reached with 3 phr filler content representing approximately 4.5% increase relative to the unfilled vulcanisate.

Not only was there no significant improvement to tensile modulus at different elongation after the saturation point; there was also a remarkable drop in modulus with MWCNT content. This development could be as a result of the high aspect ratio and high van der Waals attraction of the MWCNT. It could lead to agglomeration and limits load transfer from matrix to filler as corroborated by Ervina *et al*.^15^.

As shown in Fig. 1b, modulus at 50% elongation of NR/MWCNT nanocomposites increases with increasing MWCNT loading, except for 6 and 9 phr MWCNT fibres. The trend is better appreciated with 100%, 200% and 300% elongation where the unfilled NR has the least values of elongation at break. The results are in line with the reduction of the high elastic property of NR compound. In keeping with an earlier report by Adeosun *et al*.^16^, the nanocomposite with 3 phr showed better filler distribution and hence better crystalinity and cross-links; resulting in improved modulus. This is evident at different percent elongation reported in Fig. 1(b).

The ultimate tensile strength (UTS) of the nanocomposites and the control specimen were analysed as a function of MWCNT contents as shown in Fig. 1(c).

The UTS as a function of filler loading was carried out and an average result was reported from three different experiments. It was observed, as illustrated in Fig. 1c, that tensile strength increases with filler loading from 0 phr to 3 phr and dropped at 6 phr. This is attributed to the large specific surface area associated with CNTs as reported by Niu^17^ and an attendant increase in the cross-linking density created by vulcanisate-filler interactions^18^. Consequently, as the filler loading increases, particles tend to agglomerate thereby causing inhomogeneous dispersion of MWCNTs. The drop in tensile strength is not an uncommon phenomenon with CNT-filled nanocomposites. This could be attributed to ineffective stress transfer at particle-matrix interface as a result of poor interface adhesion^14^ and entanglement among MWCNTs^15^. It can, therefore, be concluded that the sample with 3 phr filler exhibited the highest tensile strength of 6.02 MPa; hence better capacity to withstand tensile loading.

Compressive strength as a function of MWCNTs loading is presented in Fig. 1(d). The susceptibility of the unfilled rubber to compressive loads tends to reduce with increasing filler loading. The plot takes a falling trend as filler loading increases with the compression value of the unfilled rubber matrix being the highest (approximately 38% and 46% higher than those of 15 phr and 3 phr loading respectively). This observation may not be unconnected with the reinforcing capacity of MWCNT as reported by Aguele *et al*.^19^. The adherence of the less ductile MWCNTs to the polymer phase could have stiffened the rubber chains and thus promoted resistance to compressive loading under applied strain, as established by earlier researchers^20–22^.

A departure from the trend is, however, noticed with NR/MWCNT-3. Because of the volume of MWCNTs in the sample, dispersion may have been better achieved in it compared to other samples with higher filler loading.

The data presented in Table 1 were used to compute both the mean stress and stress amplitude from each specimen. S-N testing is prepared under dynamic loading and stress. Fatigue life, at a given alternating stress level and mean stress, is the number of cycles necessary for fatigue induced failure. Therefore, going by the computation of the mean and alternating stresses, the test specimen with the highest alternating stress value (5403.40 MPa) offers the optimum fatigue life of the samples tested.

**Table 1:** Formulation of NR/MWCNTs Composites.

Sample Code						
Ingredient	NR/MWCNT-0	NR/MWCNT-3	NR/MWCNT-6	NR/MWCNT-9	NR/MWCNT-12	NR/MWCNT-15
phr^*a*^						
NR	100	100	100	100	100	100
ZnO	5.0	5.0	5.0	5.0	5.0	5.0
Stearic Acid	2.0	2.0	2.0	2.0	2.0	2.0
TMQ	1.0	1.0	1.0	1.0	1.0	1.0
MWCNTs	0.0	3.0	6.0	9.0	12.0	15.0
MBTS	1.0	1.0	1.0	1.0	1.0	1.0
Sulphur	2.5	2.5	2.5	2.5	2.5	2.5
Total	111.5	114.5	117.5	120.5	123.5	126.5

where *a*: parts per hundred parts of rubber.

However, at a cycle to failure value of 4.5 × 10^3^ computed from the S-N plots in Fig. 1(e), the alternating stress values indicate that NR/MWCNT-6 has the highest value of 9122.41 MPa followed closely by NR/MWCNT-3 (8424.41 MPa). In both cases, the unfilled vulcanisate will survive the least dynamic loading based on the computed values.

### Water absorption

**Figure 2:**
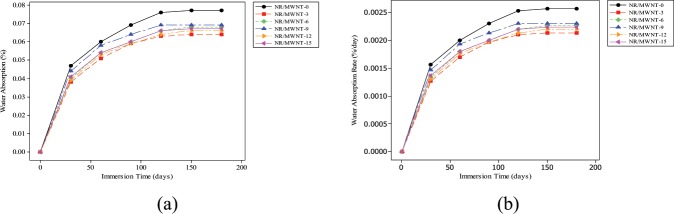
Physical properties (**a**) Water absorption (**b**) Water absorption rate.

Figure 2(a,b) show the results of water absorption and its rate respectively after 180 days of immersion in water. The weights of the specimens were taken regularly at 30 days interval. Higher initial water absorption rate was noticed for the first 30 days. This was, however, followed by a period of low but consistent water uptake in agreement with a report by Medupin *et al*.^23^. This can be attributed to diffusion phenomenon in which water molecules move from higher to lower concentration region^24^.

Water absorption generally increases with immersion time, attaining a certain value beyond which weight gain is considered insignificant; at which point the specimens have reached their saturation point. Pure vulcanisate and NR/MWCNT-9 nanocomposite showed higher values of water absorption. Of all the test specimens examined, NR/MWCNT-3 appeared to be the most stable with the least water absorption value. By the end of the first 30 days, NR/MWCNT-3 specimen has only gained approximately 0.1% of its entire weight. Although it is strange to see less filled nanocomposites to absorb more water, the water absorption profiles of the different materials indicated that even the unfilled vulcanisate absorbs relatively more water than the nanocomposites. This is an indication of good filler wetness and hence good filler-matrix adhesion in which fillers are completely embedded in the matrix phase.

### Thermogravimetric analysis

**Figure 3:**
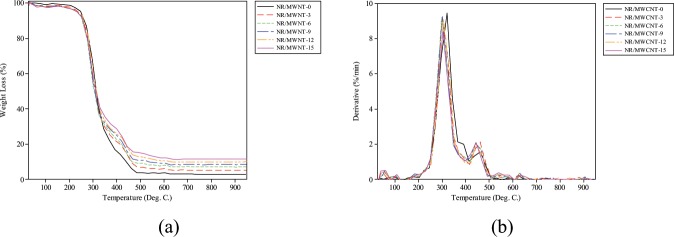
Thermogravimetric analysis (**a**) TGA micrographs of composites (**b**) DTG of composites.

The TGA and DTG of the unfilled and filled NR matrix at different MWCNT loading are presented in Fig. 3(a,b) respectively. Findings, as presented, indicate that the incorporation of MWCNTs into the NR shows considerable changes in the *T*_*onset*_ of the nanocomposites, with NR/MWCNT-3 and NR/MWCNT-12 being the most thermally stable of the formulations. The degradation profile of the NR and the resulting nanocomposites further lends credence to the assertion that the unfilled matrix is less thermally stable. This position is in synchrony with an earlier report that MWCNT reinforcement results in the enhancement of thermal stability of polymer nanocomposites^25^. The temperature range within which the nanocomposites degrade stood between 250 °C and 600 °C, beyond which there was no change of weight percentage of the nanocomposites. It can, therefore, be inferred that the thermal stability of the NR nanocomposites with regards to the unfilled vulcanisate is improved. This improvement is attributed to the combined effect of nano-confinement and barrier effect of the incorporated nanofillers^26^.

For all the nanocomposites and the unfilled NR, the first set of temperature peaks on the DTG curves (Fig. 3(b)) correspond to the initial removal of moisture and all other volatile compounds from the samples before the commencement of degradation. The temperature profile (Table 2), shows that about half of the heat required to completely eliminate moisture from the unfilled NR was needed to dehydrate NR/MWCNT-3. The moisture content was seen to also increase with MWCNT loading.

**Table 2:** Thermal parameters of NR NCs from TGA and DTG thermographs.

Samples	*T*_*inf*_ *(°C)*	*T*_*p*_ (°C)	*T*_50_ (°C)	*T*_*onset*_ (°C)	Degrad. Temp. (°C)	Residue (%)
NR/MWCNT-0	97.18	320.35	310.91	252.12	202.40–623.06	2.86
NR/MWCNT-3	45.53	299.47	307.77	260.01	202.18–416.72	5.46
					416.72–648.02	
NR/MWCNT-6	54.56	298.42	304.39	258.34	205.23–416.65	7.35
					416.65–626.20	
NR/MWCNT-9	55.88	300.16	306.58	255.70	162.48–396.07	8.64
					396.07–675.17	
NR/MWCNT-12	57.21	305.31	313.91	260.99	184.69–399.56	9.96
					399.56–655.00	
NR/MWCNT-15	59.85	303.62	310.17	258.34	187.14–396.75	11.67
					396.75–726.34	

A reason adduced for this behaviour of the nanocomposites is the depletion of the matrix phase as the filler phase predominates. It is pertinent to point out that the final residue at the end of thermal degradation increases with filler content increase in agreement with literature^27^. The residue of the unfilled rubber system is 2.86% while that of 3 phr filled system is 5.46%. The filler phase which cannot be degraded at the functional temperature of the CCVD came out as the residue after the thermal analyses of the composite. With the difference between the unfilled and 3 phr filled rubber system being 2.60%, (approximately 87% of the filler phase), the quality of mixing can be confirmed. A possible explanation for this is that as the filler concentration increases, there also increased incombustible residues which could not vapourise within the temperature range of the analysis. The increase in residue is also an indication of improved thermal stability as the filler phase predominates^28^. T_*inf*_, T_*p*_, T_50_ and T_*onset*_ are temperature values at inflection point, maximum degradation, midway of degradation process and degradation onset respectively.

Furthermore, it is clearly shown from Table 3 that the temperature at which maximum degradation occur (*T*_*p*_) for the unfilled NR drops by the incorporation of nanofillers into the matrix. This was observed to have considerably decreased over the values obtained for NR/MWCNT-12 by 4.9%. However, this is not unexpected because of the presence of the crosslinker, accelerator and activators used during the vulcanisation procedure which may have also acted as resistance to polymeric chain mobility, like MWCNTs. Therefore, the unfilled matrix is not the neat polymer normally thought of when using thermoplastics and thermosets as matrices. All of these give the vulcanisate its initial strength when compared to the raw rubber.

**Table 3:** Mixing procedure of NR/MWCNTs on two-roll mill.

Mixing order	Mixing time (minutes)
Natural rubber	4.0
ZnO, Stearic acid, TMQ	3.0
MWCNT	5.0
MBTS	1.5
Sulphur	1.5
Total time (minutes)	15.0

### SEM of nanocomposites

**Figure 4:**
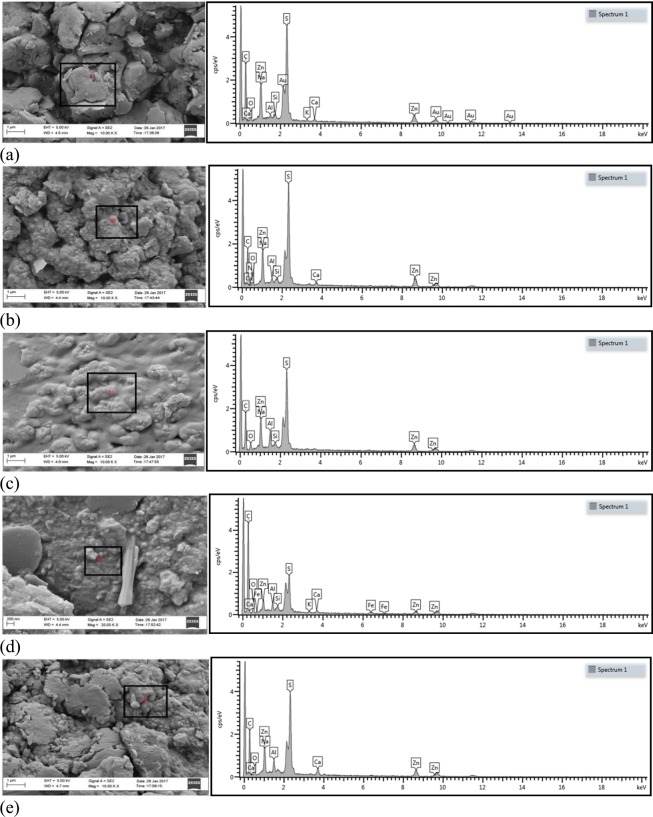
SEM microgram (left) and EDS spectrum (right) (**a**) NR/MWCNT-3 (**b**) NR/MWCNT-6 (**c**) NR/MWCNT-9 (**d**) NR/MWCNT-12 (**e**) NR/MWCNT-15.

Figure 4 shows the morphology of the tensile fractured surfaces of the test samples. It is obvious from the SEM micrographs that the filler phase were adequately embedded in the matrix phase. However, there are some holes supported by removal of the fillers which show that there is a degree of poor interaction or inadequate wetting between filler and polymer phase. At 3 phr, 6 phr and 12 phr MWCNT loading, the tensile fractured surfaces show more coarseness compared to the other nanocomposites. This, on the other hand, pointed out better interaction between the matrix and filler phases, signifying higher strength (6.02 MPa, 5.63 MPa and 5.69 MPa respectively) of the nanocomposites as can be noticed in Fig. 3. The interfacial bonding between the filler and the NR was boosted as a result of esterification mechanism and filler breakage. As a consequence, the stress was well propagated between the two phases, leading to enhanced mechanical properties.

Compositional analysis of the nanocomposites confirms the presence of the elements listed as part of the vulcanisation ingredients; MWCNTs surface treatment chemicals as well as filler elements themselves. The purification and complete functionalisation of the fillers accounts for the strong adhesive force at the interface of the two major phases which prevents filler pull-out during the fracture of the test samples in readiness for SEM/EDS analysis^29^. Investigating the elemental analysis presented in the SEM micrographs and EDS spectra, it can be pointed out that apart from the main elements suspected to have emanated from the major constituents of the NCs, other trace elements also appeared which could have come about as a result of the activities and materials used for catalyst and CNT synthesis and test sample preparation.

### Dynamic mechanical analysis

**Figure 5:**
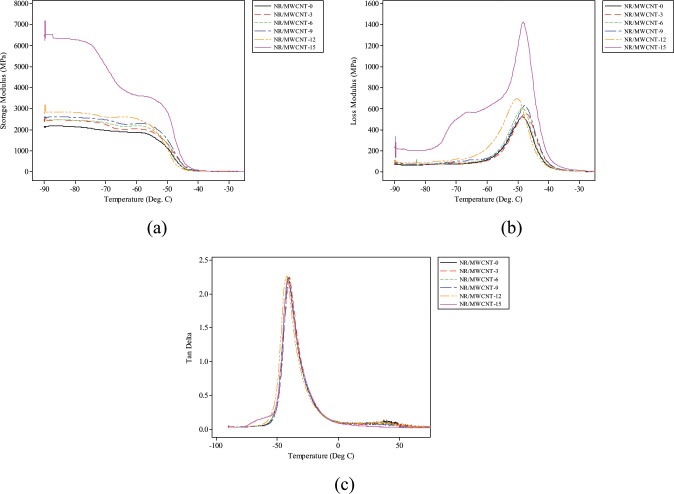
DMA Thermographs of nanocomposites (**a**) Storage modulus (**b**) Loss modulus (**c**) Tan delta.

Figure 5(a) illustrates the temperature dependence of storage modulus of the NR nanocomposites. The curves generally indicate three physical distinctive sections embodying a high modulus glassy section (ranging for 2000 MPa for the unfilled NR to over 6000 MPa for MWCNT-15 reinforced nanocomposite) where the movement of the chain segments is mostly restricted due to tight packing, prompting a high storage modulus^30,31^. In the glass transition region, storage modulus decreases significantly as temperature increases. Strong filler-matrix attractions have been suggested to be the reason behind the restriction in segmental mobility for polymer segments around the filler surface, thus causing an increase in glass transition temperature of the matrix.

It is evident from the curves that incorporation of MWCNTs engenders increase in *E*′ and this further diminishes as temperature increases. It is clear that the unfilled NR vulcanisate has the least storage modulus (2108.58 MPa) which is an indication of matrix-to-filler stress transfer according to Hedenberg and Gatenhol^32^. This value increases with increase in filler loading and peaks with 15 phr loading (6391.00 MPa). The trend is consistent with earlier studies by Chankaew *et al*.^33^ and Bras *et al*.^34^. In view of the fact that MWCNT is stiffer material than NR, the storage moduli are always higher when MWCNTs are incorporated. The downward trends in storage modulus with increase in temperature are consequent upon components becoming more mobile and lose their close packing arrangement. This leads to such a noticeable decrease in *E*′ in the glass transition region.

However, since the newly developed materials are intended to be used at room temperature and probably slightly above it, it was necessary to investigate their stiffness level at temperatures mostly encountered. The storage modulus of NR/MWCNT-15 is about 600% higher than other filled NCs at temperatures from 32 °C to 37 °C, averaging 0.42 MPa for the unfilled vulcanisate which has the least value. This value was seen to increase with the incorporation of MWCNTs. With such a high average storage modulus of 6.11 MPa, NR/MWCNT-15 is considered the stiffest of the different composition.

The storage modulus results is consistent with an earlier report by Wu *et al*.^30^ that there was no evidence of reinforcement with filler loading at lower temperature; but rather a reduction in *E*′. This study presents a considerable decrease in *E*′ as temperature increases with significant decrease in the range between −85 °C and −42 °C corresponding to glass transition region. The drastic decrease is attributed to the increase in the chain mobility of the NR near the glass transition region.

The loss modulus, *E*″, values which reflect the amount of viscous dissipation in the NCs are presented in Fig. 5(b). When plotted against temperature, the peak values of *E*″ gives the glass transition temperatures (*T*_*g*_) values of the nanocomposite systems. It can be reported that *T*_*g*_ of the nanocomposites decreases with the addition of the nanotubes into the NR vulcanisates. This is contrary to earlier reports where other inorganic micron-sized fillers were used^35^, but consistent with a recent work by Sagar *et al*.^27^. *β* relaxation is the most conspicuous peak observed for both neat NR vulcanisate and nanocomposites all through the temperature range used in the experiment. It was suggested in Behzad *et al*.^36^ that a clear *β* relaxation peak associated with the relaxation of the branched points in non-crystalline material is attributed to the segmental motions of the phase. This also corresponds to the glass transition temperature in NR whose *T*_*g*_ falls in the sub-zero region of the temperature axis.

The obvious closeness of *T*_*g*_ for each of the materials indicates that they can be operated at the same working condition and that reinforcement has little or no effect on the glass transition temperature of elastomeric composites. The lower temperature transition of *E*″ as the filler loading increased disagrees with an earlier work by Lafia-Araga *et al*.^37^ and Padal *et al*.^38^ in which micron-scaled organic materials were used as reinforcing fillers in a thermoplastic matrix.

A percentage increase of about 300% is observed for composites loaded with 15 phr MWCNTs in comparison to unfilled NR. The high loss modulus (1413 MPa) at this temperature (−50.43 °C) can be attributed to high concentration of MWCNT filler which increased the stiffness of the material by establishing restriction on the segmental mobility of the polymer chains at the relaxation temperature^39^. However, because of the area of application in this study, increased stiffness beyond those provided by the 3 phr filler loading may be undesirable. This is because it is practically impossible to achieve optimum homogeneous filler distribution beyond 5 phr filler loading owing to high tendency of MWCNTs to agglomerate at higher reinforcement concentration^40^.

The tan δ of the nanocomposites and the pure vulcanisate is displayed in Fig. 5(c). Tan δ usually occurs in the glass transition region and related to the movement of low molecular weight unit and molecular chains within the rubbers. Unlike the case of the E″ in which the composites present higher amplitude peaks as compared to the pure vulcanisate, it can be seen that tan δ of the composites and pure vulcanisate are in close proximity. The findings indicate that the chain mobility of the matrix reduced significantly with increased filler loading. This reduced chain mobility is attributed to both physical and chemical adsorption of matrix molecules on the filler surface which results in height reduction of tan δ peaks during dynamic mechanical deformation^41^.

The tan δ curves show narrow peaks which points to the reinforcing efficiency of the MWCNTs, characteristic of complete crosslink process of the NR matrix formulations^1,42^. These narrow peaks noticed for the different samples further validate the production route adopted for the manufacture. In another study by Padal *et al*.^38^, the height of tan δ curves was said to be directly connected to the materials’ capacity to dissipate energy through segmental motion. Composite systems with higher tan δ peaks have higher ratios of energy absorption, viscous motions and are tougher than those with lower tan δ amplitudes. It is against this background that 3 phr loading remains the optimum filler loading for nanocomposite system suitable for prosthetic application, as evidenced in the test samples reported.

## Wear Rate

Wear resistance test of NR/MWCNT-3 and an existing artificial foot (EAF) material was carried out. The decision to single NR/MWCNT-3 is consequent upon its unique and superior properties over the other nanocomposite materials studied. The results are presented in Table 4.

**Table 4:** Wear Rate Results.

sample	Initial mass (g)	final mass (g)	Density (g/cm^3^)	Mass loss (g)	volume loss (cm^3^)	Sliding Dist. (m)	Wear Rate (cm^3^/Nm)
NR/MWCNT-3	3.577	3.561	0.36	0.016	0.044	4.45	0.001977
EAF	3.107	3.089	0.31	0.018	0.058	4.01	0.002896

It can be seen from the table that NR/MWCNT-3 has a lower mass loss when compared to the earlier foreign artificial foot. It can, therefore, be concluded that NR/MWCNT-3 offers better options in wear resistance since materials of higher abrasion resistance are reported to have a lower volume loss according to Agarwal *et al*.^43^.

## Conclusion

This study is focused on the formulation of NR/MWCNT nanocomposite for anthropomorphic prosthetic foot application. It can be concluded that the combined actions of chemical purification of MWCNTs and mechanical mixing of the same with the matrix phase, in the right proportion, resulted in the improved properties of the nanocomposites. Of the five different formulations studied, NR/MWCNT-3 exhibited better capacity to withstand tensile loading, offers the optimum dimensional stability at ambient conditions and is considered the most eco-friendly of the materials with water absorption approximately 0.1%. Finally, wear rate test of NR/MWCNT-3 as compared to an existing prosthetic foot material revealed that the material possesses better wear resistance ability and has the highest capacity to dissipate energy through segmental motion. Improving its aesthetic acceptability among users by discovering the appropriate colouring agent that will not impact negatively of the inherent properties of the filler material is recommended for future works.

## Materials and Methods

### Materials

The polymeric material used for this study is natural rubber (*Hevea brasiliencis*). MWCNTs were synthesised using VACUTEC chemical vapour deposition equipment (model: XD-1200NT, USA) at the Centre for Genetic Engineering and Biotechnology, Federal University of Technology, Minna, Nigeria. Stearic acid, sulphur, zinc oxide (ZnO), 2–2-dithiobis benzothiazole (MBTS), and 2,2,4-Trimethyl-1,2-dihydroquinoline polymer (TMQ) were received from the Nigeria Institute of Leather Science and Technology, Zaria, Nigeria while sodium dodecylbenzene sulfonate (C_18_H_29_NaO_3_S), nickel (II) nitrate hexahydrate, Ni(NO_3_)_2_.6H_2_O, with molecular weight of 290.79 g/mol, iron (III) nitrate nonahydrate, Fe(NO_3_)_3_.9H_2_O, with molecular weight of 403.99 g/mol, and aluminium oxide, Al_2_O_3_, with molar mass 101.96 g/mol were supplied by Sigma-Aldrich with percentage purity ranging from 95.0% to 99.9%.

### Methodology

#### Compounding of natural rubber matrix composite

Compound formulation followed the procedure described by Hemkaew *et al*.^44^. Natural rubber compounds were prepared at 65 ± 2 °C temperature in a two-roll open mill (5183, USA) with the speed of the front and rear rolls adjusted to 30 rpm and 18 rpm respectively.

The formulations used for preparing the rubber nanocomposites are illustrated in Table 1. Mixing was performed according to ASTM D-3182 standard on a two-roll mill with 300 mm working distance and outside diameters of 115 mm with a gear ratio of 1. The vulcanisation process was accelerated using MBTS which is considered one of the safest of the currently used accelerators^45^ in order to reduce vulcanising time. Zinc oxide (ZnO) and stearic acid were used as activators to increase the effectiveness of the accelerator. Combining ZnO with stearic acid helped to reduce the time of vulcanisation and also to improve wear resistance of the base elastomer, and in consequence, the nanocomposites. TMQ served as the antioxidant in the system. Table 3 shows the sequence of addition of vulcanisation ingredients. MBTS and sulphur were added last with the former coming earlier to accelerate the process.

After compounding the different formulations, they were left for 24 hours at ambient conditions to allow for full interaction of the ingredients. They were then cured at 150 ± 2 °C for 10 minutes in an electrically heated hydraulic press. Test samples were then cooled under the curing pressure of 0.2 MPa at room temperature and neatly packed for characterisation.

### Characterisation of the composites

#### Mechanical test

The tensile test specimens were prepared consistent with ASTM D638 standard and notching or cracks were avoided. Instron machine was used for the test. The system was set up by inputting the necessary information of gauge length (22.50 mm), thickness (2.8 mm) and width (6.00 mm) of the specimen.

Compression test was carried out according to ASTM D 3410/D 3410M-03 standards under room temperature. The test specimen was inserted into the fixture which is placed between the platens of the testing machine and then compression loaded. The ultimate compressive stress of the materials, as obtained with the test fixture and specimens, can be obtained from the maximum force carried out before failure. Three runs of tests were carried out and the average values were reported.

Fatigue test was conducted according to ASTM E606/E606M-12 standard under room temperature using a 300 kgFcm capacity fatigue test machine (Avery Denison 7305). The number of cycles to failure was recorded by the revolution counter fitted to the motor. The failure of the specimen was indicated by the abrupt cut out of the switch and the attendant automatic stop of the machine.

The maximum and minimum stresses are computed using Eqs. 1 and 2.

1 $${\rm{\sigma }}={{\rm{M}}}_{{\rm{\max }}}/{\rm{W}}\,$$

2 $${\rm{W}}={{\rm{\pi }}{\rm{d}}}^{3}/32\,$$

where ${M}_{max}$ is the bending moment of the test specimen which was read directly from the fatigue equipment and *W* is the section modulus. The mean stress, ${\sigma }_{m}$, which is the average level of a constant amplitude cyclic loading and the stress amplitude, ${\sigma }_{a}$, is the variation about this mean are also computed.

Abrasion wear test was conducted using TR-50 Dry Abrasion Tester in accordance to ASTM G65 standard. The specified force of the test was 5 N. Mass loss of test samples indicated that wear has taken place. The material of higher abrasion resistance has a lower mass loss.

Mass loss was calculated using Eq. 3.

3 $$\varDelta m={m}_{1}-{m}_{2}$$

where $\Delta m$ is the mass loss of specimen; ${m}_{1}$ and ${m}_{2}$ are masses of the specimens before and after test respectively.

Volume losses of the specimens were computed in the following way:

4 $$\Delta V=(\varDelta V/\rho )\times 1000\,$$

where $\rho$ is the experimental density of the specimen.

The specific wear rates of the specimens were calculated thus:

5 $${W}_{s}=\varDelta V/({F}_{n}\times {S}_{s})\,$$

where ${W}_{s}$ is the wear rate of the specimen, ${F}_{n}$ is the normal load applied of the specimen and ${S}_{s}$ is the sliding distance through which the specimens were traversed.

### Water immersion tests

Five specimens for each of the nanocomposite formulation were dried in an oven at 70 °C for 6 hours in order to drain the moisture in them in preparation for water absorption test carried out according to ASTM D-7031–04 standard. The weight of each specimen was determined to a precision of 0.001 g and immersed in distilled water at room temperature according to a method adopted by Mohebby *et al*.^46^. Wet weights of the samples immersed in water were measured within 6 months soaking period at room temperature. Water absorption as well as their rate was determined according to the relationship presented in Eqs. 6 and 7.

6 $$M{C}_{t}=({M}_{t}-{M}_{o})/{M}_{o}\times 100\,$$

where $M{C}_{t}$ is water absorption at time t (%), ${M}_{t}$ is wet weight at time t (g) and ${M}_{o}$ is the dried weight (g).

7 $$Rate\,( \% /hr)=\Delta X/\Delta t=({X}_{{t}_{i}}-{X}_{{t}_{i-1}})/({t}_{i}-{t}_{i-1})$$

where $Rate\,( \% /hr)$, ${X}_{{t}_{i}}$ and ${X}_{{t}_{i-1}}$ are absorption or swelling in immersed condition at time ${t}_{i}$ and ${t}_{i-1}$.

#### Thermal analysis

Thermal degradation test was run in line with global mass loss by using Perkin Elmer TGA. About 25 mg of the sample was spread on a 7.4 mm diameter open sample pan and 4.2 mm deep. The temperature change was controlled from 30 °C to 950 °C at a heating rate of 10 °C/min. The sampling segment was set as 0.5 second per point.

The samples’ response to external cyclic stress was observed using TA Instruments, DMA Q800. They were subjected to temperature ramp from −90 °C to +100 °C at a rate of 3 K/min and frequency of 1 Hz. The operation was carried out in the dual cantilever bend mode with a dynamic force of 2.5 N and amplitude of 15.0 µm. Storage modulus and mechanical damping of the nanocomposites were measured as a function of the temperature in the DMA test. Following the mounting and cooling of the samples by liquid nitrogen for about 30 minutes to temperature of –90 °C, the storage and loss modulus and damping curves were generated and recorded.

#### Wear rate test

In accordance to ASTM G65 standard, abrasion wear test was conducted using TR-50 Dry Abrasion Tester. The specimen (NR/MWCNT-3) was firmly held in the holder and normal load was applied to it with the face of the specimen in contact with a rotating rubber wheel. The test specimen was pressed against the rotating wheel at a specified force of 5 N using a lever arm while a restricted flow of grit abraded the surface of the test specimen. The wheel rotated in such a way that the contact faces move in the direction of the sand flow. Weight loss of test sample indicated that wear has taken place.

The specific wear rates of the specimens were calculated using Equations adopted from the works of Agarwal *et al*.^43^ and Vemuri and Vincent^47^.

## Supplementary information

Supplementary information.
