# Supplementary material for: Carbon Nanotube Reinforced Natural Rubber Nanocomposite for Anthropomorphic Prosthetic Foot Purpose
Source: Sci Rep. 2019 Dec 27;9:20146. doi: 10.1038/s41598-019-56778-0 (PMC6934845; doi:10.1038/s41598-019-56778-0)
Supplement: Supplementary file 1 — Supplementary information. [file 41598_2019_56778_MOESM1_ESM.pdf]

Carbon Nanotube Reinforced Natural Rubber Nanocomposite for Anthropomorphic Prosthetic Foot Purpose

Rasaq Olawale Medupin[1,4,*], Oladiran Kamardeen Abubakre[2,4,+], Ambali Saka Abdulkareem[3,4,+], Rasheed Aremu Muriana[2,4,+] and Asipita Salawu Abdulrahman[2,4]

[1]The Federal Polytechnic, P. M. B. 55, Department of Mechanical Engineering, Bida, Nigeria

[2]Federal University of Technology, P. M. B. 56, Department of Metallurgical & Materials Engineering, Minna, Nigeria

[3]Federal University of Technology, P. M. B. 56, Department of Chemical Engineering, Minna, Nigeria

[4]Federal University of Technology P. M. B. 56, Centre for Genetic Engineering and Biotechnology, Minna, Nigeria

Corresponding Author's e-mail: *medupin.pg11723@st.futminna.edu.ng*

Supplementary Information

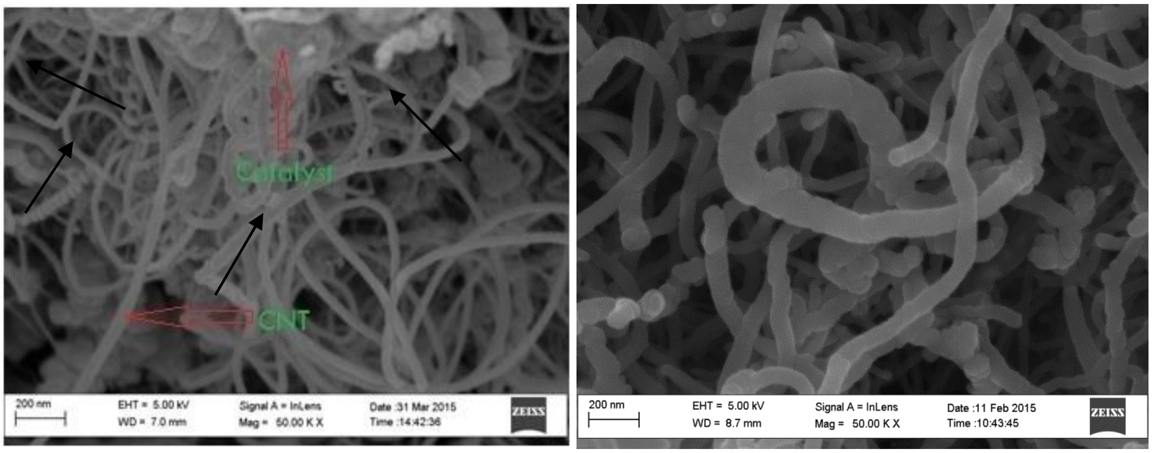

Supplementary Fig. S1: HRSEM micrograph of as-produced (left) and purified (right) MWCNTs used for reinforcement

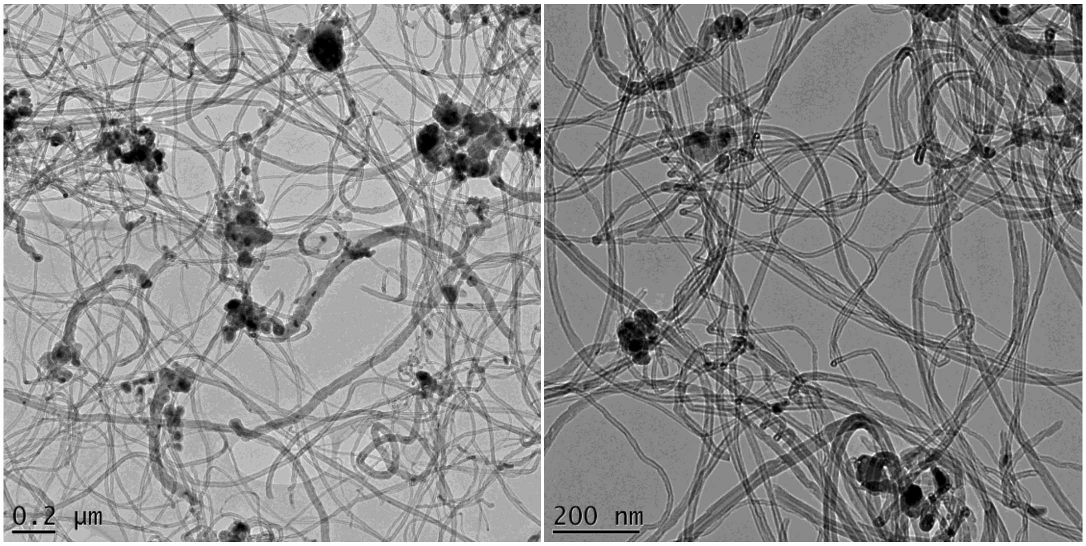

Supplementary Fig. S2: HRTEM images of as-produced (left) and purified (right) MWCNTs used for reinforcement

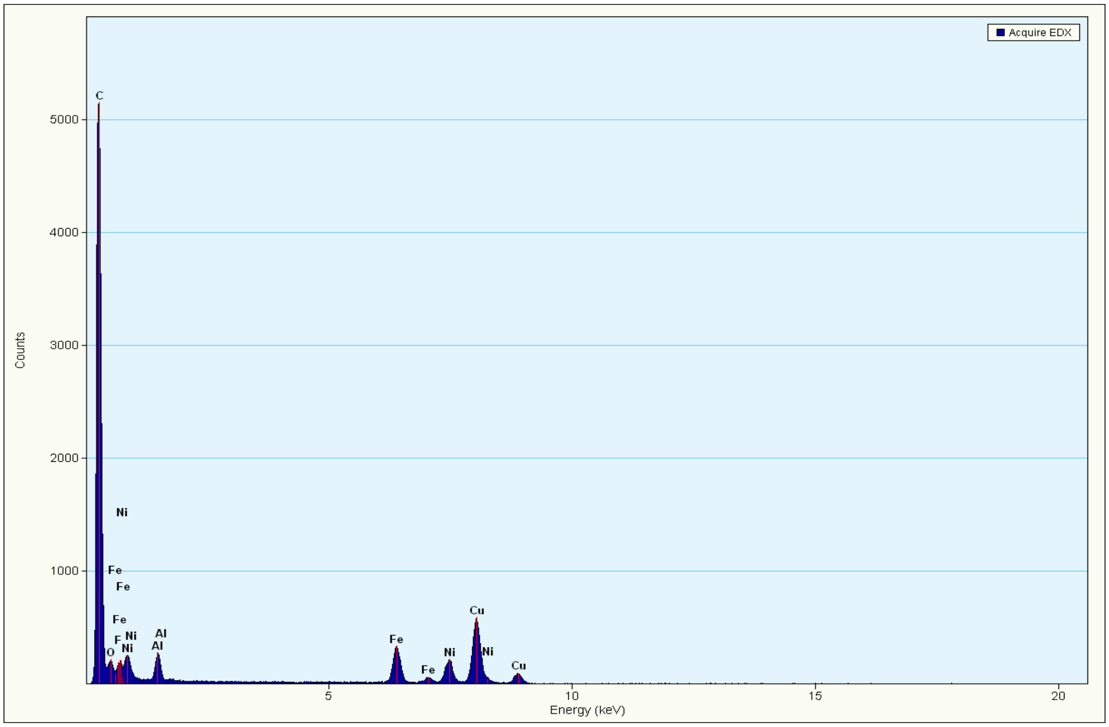

Supplementary Fig. S3: EDX of purified MWCNT

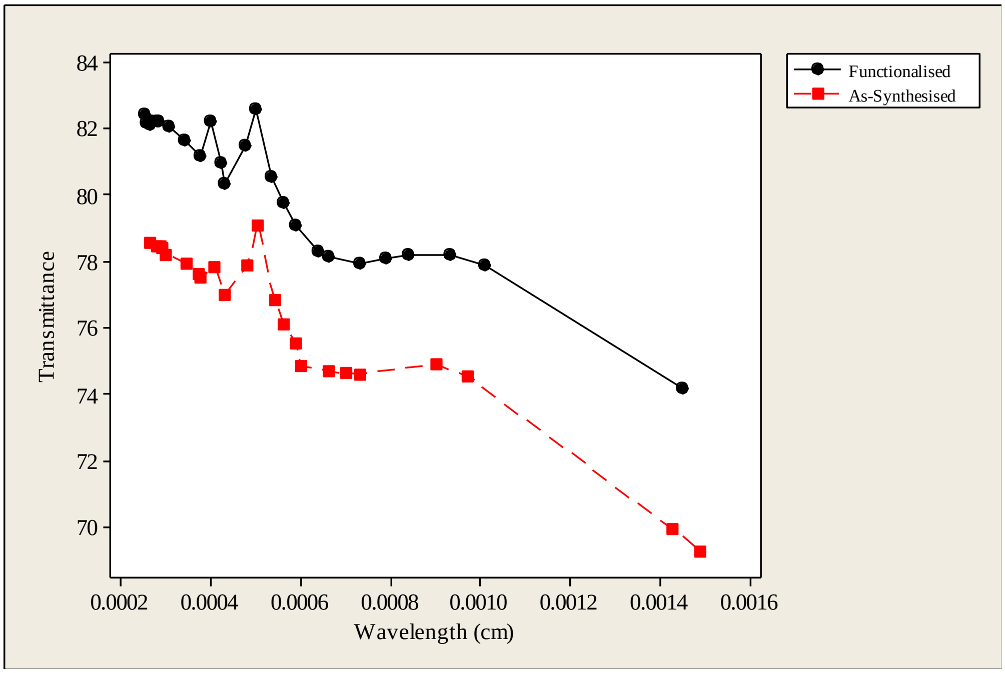

Supplementary Fig. S4: FTIR spectra of as-synthesised and functionalised MWCNTs

Supplementary Table S1: DMA data of nanocomposites and control test samples

Nanocomposites	Peak height of tan δ curve	T_g from tan δ_{max} (ºC)	Peak height of E'' curve	T_g from E''_{max} (ºC)
NR/MWCNT-0	2.219	-41.61	516.11	-48.91
NR/MWCNT-3	2.239	-42.42	550.50	-49.57
NR/MWCNT-6	2.229	-43.95	607.10	-51.01
NR/MWCNT-9	2.150	-42.98	624.40	-50.08
NR/MWCNT-12	2.243	-45.15	691.50	-52.82
NR/MWCNT-15	2.187	-43.78	1413.00	-50.43

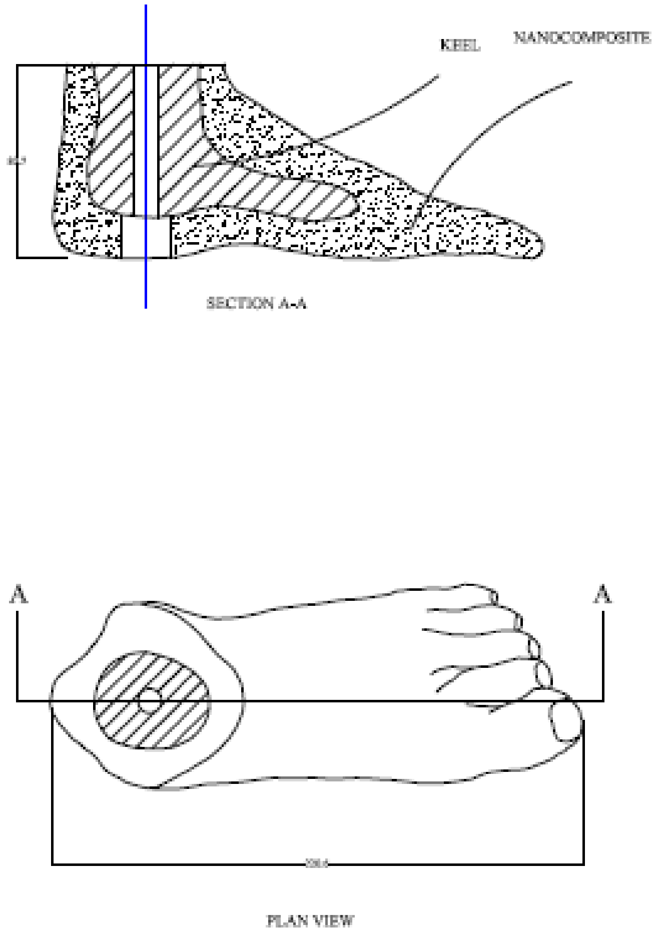

Sectional view of the Prosthetic foot
